# Comparison of TPF and TP Induction Chemotherapy for Locally Advanced Nasopharyngeal Carcinoma Based on TNM Stage and Pretreatment Systemic Immune-Inflammation Index

**DOI:** 10.3389/fonc.2021.731543

**Published:** 2021-09-20

**Authors:** Ying Xiong, Liangliang Shi, Lisheng Zhu, Gang Peng

**Affiliations:** Cancer Center, Union Hospital, Tongji Medical College, Huazhong University of Science and Technology, Wuhan, China

**Keywords:** locally advanced nasopharyngeal carcinoma, induction chemotherapy, systemic immune-inflammation index, prognosis, toxicity

## Abstract

**Purpose:**

To evaluate the efficacy and toxicity of the two IC (induction chemotherapy) regimens, TPF (taxanes, cisplatin, and 5-fluorouracil) and TP (taxanes and cisplatin) combined with concurrent chemoradiotherapy (CCRT) in locally advanced nasopharyngeal carcinoma (LA-NPC) patients.

**Methods:**

Ultimately, we enrolled 213 patients at stage III-IVA in this retrospective study. The prognosis of TPF and TP was compared by Kaplan-Meier and Cox proportional hazard regression. The toxicities were evaluated according to CTCAE v4.0 and RTOG criteria.

**Results:**

TPF was found to have a higher 5-year DMFS in stage IVA and N2-3 patients. The optimal value of pretreatment SII was 432.48. A further subgroup analysis revealed that patients in stage IVA combined with SII ≥432.48 showed superior OS (*P*=0.038) and DMFS (*P*=0.028) from TPF. Also, SII was proved to be a prognostic element for PFS (HR 2.801, P=0.018) and DMFS (HR 3.735, P=0.032) in multivariate analysis, and IC regimen (HR 2.182, P=0.049) for predicting DMFS. The rate of grade 3–4 leukopenia (*P*=0.038), neutropenia (*P*=0.021), radiation oral mucositis (*P*=0.048), diarrhea (*P*=0.036), and ear damage (*P*=0.046) were more common in TPF group.

**Conclusion:**

Our study revealed that TPF regimen showed a higher 5-year DMFS for stage IVA and N2-3 patients, while for stage III and N0-1, TP might be ample. In high-risk LA-NPC patients (stage IVA combined with pretreatment SII ≥432.48), TPF had a higher 5-year OS and DMFS, with more grade 3–4 toxicities, but most of them were endurable.

## Introduction

Nasopharyngeal carcinoma (NPC) is a malignant head and neck tumor that occurs at the top and lateral wall of the nasopharyngeal cavity, with a relatively higher incidence in China and Southeast Asia and 129,000 new cases diagnosed worldwide (1). Early symptoms are hidden, and 75% patients have been diagnosed with NPC at stage III or IVA. Due to its special anatomy and sensitivity to radiation, concurrent chemoradiotherapy (CCRT) is regarded as the main treatment in locally advanced NPC (LA-NPC). As intensity-modulated radiotherapy (IMRT) improving, local control rates of LA-NPC were improved; however, distant metastasis still remains a major failure pattern.

Accumulating studies confirms that induction chemotherapy (IC) could help to control subclinical micrometastasis (2). A phase III trial (3) showed that IC followed by CCRT could improve overall survival (OS), distant metastasis-free survival (DMFS), and disease-free survival (DFS) in LA-NPC when compared with CCRT. A recent study (4) showed that IC plus CCRT could increase OS (*P*<0.001), PFS (*P*<0.001), DMFS (*P*<0.001), and LRFS (*P*<0.001) in LA-NPC. Similarly, the survival benefits brought by IC followed by CCRT have been confirmed in many other studies (5). As a result, based on the National Comprehensive Cancer Network (NCCN) guideline, IC followed by CCRT is suggested in the category 1A recommendations for LA-NPC (6).

As we know, the first-line IC regimens including Docetaxel, cisplatin, and 5-fluorouracil (TPF), Docetaxel and cisplatin (TP), cisplatin and 5-fluorouracil (PF), Gemcitabine and cisplatin (GP) have brought some survival advantages in studies (7). At present, TPF is the main regimen, but accompanied by its long treatment time and adverse reactions caused by 5-FU, such as myelosuppression and diarrhea. Therefore, it is crucial whether the TP regimen can reduce the related toxicities while ensuring the survival benefit. A previous research performed by Wang et al. (8) in LA-NPC showed that, TPF (docetaxel 60 mg/m^2^, cisplatin 25 mg/m^2^, days 1–3, 5-FU 500 mg/m^2^, days 1–3) had similar efficacy compared to TP, and the grade 3–4 toxicity in TP group is lower, which provided an idea for TP regimen as an alternative to TPF. However, the standard dose of 5-FU was lowered as considering the tolerance of patients, so we could not completely rule out the potential effect of dose. At present, there is still no consensus about the efficacy and safety of the two regimens. Therefore, this paper was conducted to compare the survival efficacy and treatment-related toxicity of TPF and TP regimen in LA-NPC patients, in order to explore the feasibility of alternative TP regimen.

In addition, the TNM staging system is still considered as the reference standard for evaluating the survival in patients, but the prognosis of patients who received similar treatment in the same period is different, as the internal tumor heterogeneity is not taken into account by TNM staging. Nowadays, accumulating evidence have shown that inflammation contributes to the development, growth, and metastasis of cancer cells (9). And systemic immune-inflammation index (SII), a new hematological index, has been identified as a prognostic biomarker in NPC (10). It is worth pointing out that patients with NPC in our analysis were divided into different subgroups according to the pretreatment SII levels, which was not reported in previous studies.

## Materials and Methods

### Patients

A total of 213 patients diagnosed with LA-NPC at Union Hospital Cancer Center from January 2013 and December 2017 were enrolled. The inclusion criteria were as follows: (1) pathologically verified NPC at the first diagnosis; (2) Karnofsky performance status (KPS) ≥70; (3) age between 16 and 70 years; (4) a complete examination, including nasopharyngeal speculum, lung CT, enhanced MRI of the nasopharynx and neck, abdominal ultrasound, and a whole-body bone scan (or whole-body PET-CT), and finally re-staged as III-IVA according to the 8th edition of the AJCC staging system; and (5) complete data of hematological parameters, including neutrophil, lymphocyte, and platelet counts within 7 days before treatment. The exclusion criteria were as follows: (1) a history of second primary malignant tumor; (2) a history of anticancer therapy; (3) an unfinished IC followed by CCRT; (4) a poor function of heart, lung, liver, and renal; and (5) complicated with acute infection or autoimmune diseases. Written consent was obtained from enrolled patients, and the study was approved by Cancer Center of Union Hospital of Tongji Medical College of Huazhong University of Science and Technology.

### Methods

IMRT was conducted with 6MV X-ray linear accelerator. And principles of target delineation are as follows: Gross tumor volume of the nasopharynx (GTVnx): 66–76 Gy/33F; Gross tumor volume of the positive neck lymph nodes (GTVnd): 66–70 Gy/33F; Clinical target volume 1 (CTV1): 60–66 Gy/33F; Clinical target volume 2 (CTV2): 54–60 Gy/33F. The fractionated dose was 1.8 to 2.2 Gy at one fraction per day and 5 days per week. PTV (Planning target volume) was expanded by adding 3 mm to the GTV and CTV, respectively. The IC regimens were as follows: (1) TPF regimen: docetaxel (75 mg/m^2^/day, day 1), cisplatin (75 mg/m^2^/day, day 1), and 5-fluorouracil (750 mg/m^2^/day, days 1–5); and (2) TP regimen: docetaxel (75 mg/m^2^/day, day 1) and cisplatin (75 mg/m^2^/day, day 1). IC were conducted every 21 days for three cycles. Besides, the cumulative dose of cisplatin during the concurrent chemotherapy was 200 mg/m^2^.

### Data Collection and Clinical Endpoints

The clinical data of all patients before treatment were collected were sex, age, smoking and drinking history, EBV-DNA status, T stage, N stage, clinical stage, and IC regimen. Hematological data before treatment were peripheral blood neutrophils, lymphocytes, and platelet count. SII is defined as total platelet count (10^9^/L) × neutrophil count (10^9^/L)/total lymphocyte count (10^9^/L). The follow-up data: the time of beginning of follow-up, death, disease progression, and the deadline of follow-up.

The endpoints were as follows: OS, defined as the time from pathological diagnosis to death of any cause or the last follow-up; Progression-free survival (PFS), the time from pathological diagnosis to tumor progression or death for any cause; Locoregional relapse-free survival (LRFS), the time from pathological diagnosis to local recurrence; DMFS, the time from the pathological diagnosis to the distant metastasis.

Treatment-related side effects between the groups were evaluated according to CTCAE V4.0 (Common Terminology Criteria for Adverse Events V4.0) (11) and RTOG (Radiation Therapy Oncology Group) criteria (12).

### Follow-Up

The frequency of follow-up after treatment was every 3 months in the first 2 years, every 6 months in the 3 to 5 years, and then annually after 5 years. The follow-up included complete medical records. All patients were followed up by each clinical examination in the hospital or telephone calls.

### Statistical Analyses

SPSS 25.0 and GraphPad Prism 8.0 software were used to analyze the data. The optimal cutoff value of SII was decided according to the receiver operating characteristic (ROC) curve. The measurement data were tested by independent sample t-test or Mann-Whitney U test, and the classified variables were tested by chi-square test. Survival curves were analyzed by Kaplan-Meier method and univariate analysis by Log-rank. Cox proportional hazard regression model was adopted in multivariate analysis. *P* value less than 0.05 was considered as statistically significant.

## Results

### Baseline Characteristics and Follow-Up

**Figure 1:**
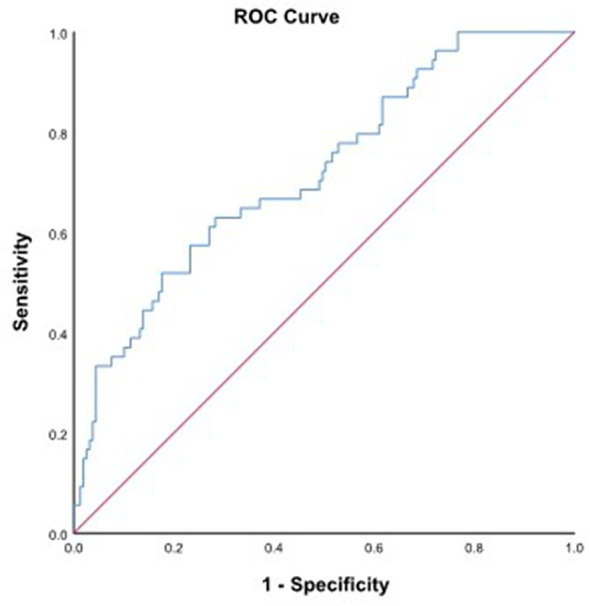
ROC curve for pretreatment SII = 432.48 based on OS (*P*=0.011, Sensitivity: 95.0%, Specificity: 34.7%, AUC=0.673). ROC, receiver operating characteristic; SII, systemic immune-inflammation index; OS, overall survival.

Ultimately, 213 patients diagnosed at stage III-IVA were enrolled, with 128 and 85 patients in the TPF and TP group, respectively, whose baseline characteristics are shown in **Table 1**. Among them, 155 (72.77%) were males and 58 (27.23%) were females, with a median age of 45 years. One hundred one (47.42%) and 87 (40.85%) patients had a history of smoking and drinking, respectively. In the cohort, 121 (56.81%) patients were diagnosed with positive EBV DNA status. Based on the TNM staging system, 115 (53.99%) and 98 (46.01%) patients were re-staged in stage III and IVA, respectively. According to the ROC curve, the optimal cutoff value of pretreatment SII was 432.48 (*P*=0.011, Sensitivity: 95.0%, Specificity: 34.7%, AUC=0.673) (**Figure 1**), with 67 (31.46%) cases in low SII group (SII < 432.48) and 146 (68.64%) cases in a higher SII group, respectively.

**Table 1:** Baseline characteristics of patients in the TPF and TP groups.

Variables	TPF (n = 128) (%)	TP (n = 85) (%)	*P*
**Age (years)**			0.166
<45	38 (29.69)	33 (38.82)	
≥45	90 (70.31)	52 (61.18)	
**Sex**			0.127
Female	30 (23.44)	28 (32.94)	
Male	98 (76.56)	57 (67.06)	
**Smoke**			
No	60 (46.88)	52 (61.18)	0.051
Yes	68 (53.12)	33 (38.82)	
**Drink**			0.290
No	72 (56.25)	54 (63.53)	
Yes	56 (43.75)	31 (36.47)	
**EBV DNA status**			0.628
Negative	57 (44.53)	35 (41.18)	
Positive	71 (55.47)	50 (58.82)	
**Tumor classification**			0.406
T1	1 (0.78)	0 (0.00)	
T2	18 (14.06)	18 (21.17)	
T3	56 (43.75)	38 (44.71)	
T4	53 (41.41)	29 (34.12)	
**Nodal classification**			0.213
N0	2 (1.56)	3 (3.53)	
N1	30 (23.44)	15 (17.65)	
N2	78 (60.94)	47 (55.29)	
N3	18 (14.06)	20 (23.53)	
**Clinical stage**			0.756
III	60 (46.88)	38 (44.71)	
IVA	68 (53.12)	47 (55.29)	
**Pretreatment SII level**			0.824
<432.48	41 (32.03)	26 (30.59)	
≥432.48	87 (67.97)	59 (69.41)	

TPF, docetaxel, cisplatin, and 5-fluorouracil; TP, docetaxel and cisplatin; EBV DNA, Epstein-Barr virus DNA; SII, systemic immune-inflammation index.

As shown in the table, there was no significant difference in the two regimen groups (*P* > 0.05). In whole, the follow-up time ranged from 26 to 83 months. Finally, 20 (9.39%) patients died, and 54 (25.35%) patients suffered from tumor progression, of which 28 (51.85%) and 26 (48.15%) patients had local progression and distant metastasis, respectively. The 5-year OS, PFS, LRFS, and DMFS rates in TPF and TP groups were 89.0 *vs* 82.4%, 76.8 *vs* 68.4%, 85.9 *vs* 86.9% and 90.2 *vs* 81.3%, respectively.

### Survival Analysis Based on TNM Staging System

**Figure 2:**
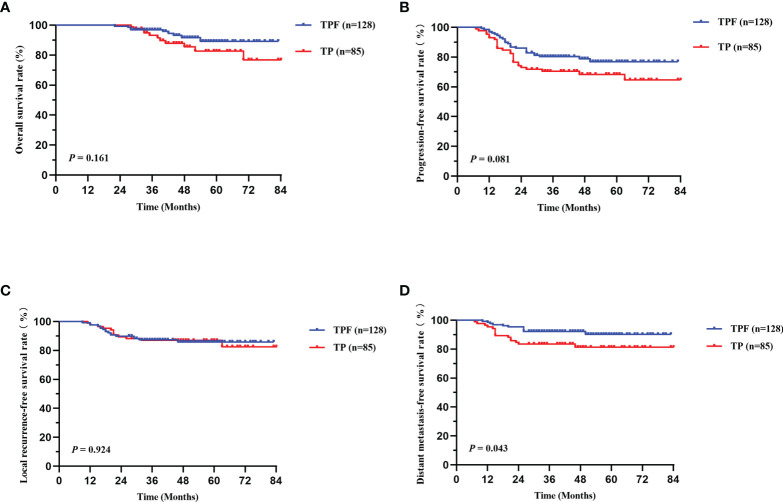
Kaplan-Meier survival curves of OS **(A)**, PFS **(B)**, LRFS **(C)**, and DMFS **(D)** between TPF and TP groups in locally advanced patients. TPF, docetaxel, cisplatin, and 5-fluorouracil; TP, docetaxel and cisplatin; OS, overall survival; PFS, progression-free survival; LRFS, locoregional relapse-free survival; DMFS, distant metastasis-free survival.

Survival curves based on the different IC regimens were analyzed using the Kaplan-Meier method. As was shown in **Figure 2**, the patients in TPF group showed superior 5-year DMFS (90.2 *vs* 81.3%, *P* = 0.043, **Figure 2D**). However, no evident difference was found in OS, PFS, and LRFS between the two groups (*P* > 0.050).

**Figure 3:**
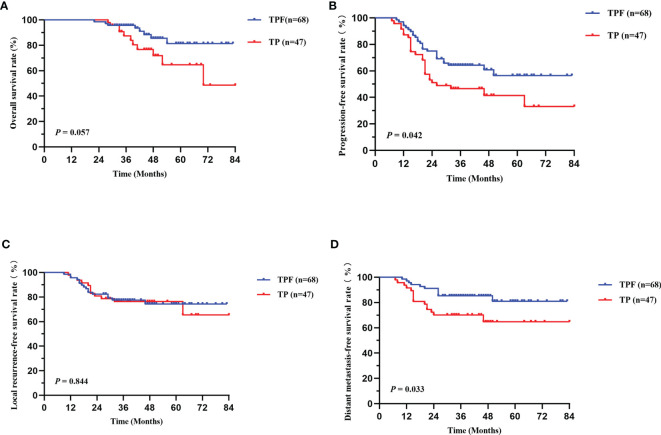
Kaplan-Meier survival curves of OS **(A)**, PFS **(B)**, LRFS **(C)**, and DMFS **(D)** between TPF and TP groups in patients with stage IVA. TPF, docetaxel, cisplatin, and 5-fluorouracil; TP, docetaxel and cisplatin; OS, overall survival; PFS, progression-free survival; LRFS, locoregional relapse-free survival; DMFS, distant metastasis-free survival.

Patients in different TNM stages showed different tumor load and treatment failure rate. Therefore, survival differences among patients in different clinical and N stage subgroups were conducted separately, with 98 in stage III and 115 in stage IVA. Since only five stage N0 patients were included, in order to minimize the deviation of statistical analysis, we divided N stage into N0-1 and N2-3 subgroups, including 50 and 163 cases, respectively. As shown in **Figure 3**, no significant survival difference was found in stage IVA patients between the two groups, and the TPF group had superior PFS (*P* = 0.042, **Figure 3B**) and DMFS (*P* = 0.033, **Figure 3D**). Similarly, we found that stage N2-3 patients in TPF also showed a significant trend in a higher DMFS (*P* = 0.057, **Supplementary Figure 3**). However, in patients with stage III and N0-1, no survival difference was found (*P* > 0.050, **Supplementary Figure 3**).

### Survival Analysis in Stage IVA Patients Combined With Pretreatment SII

**Figure 4:**
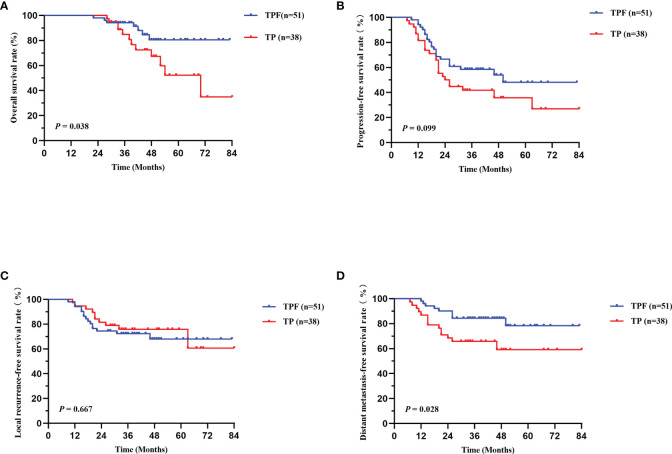
Kaplan-Meier survival curves of OS **(A)**, PFS **(B)**, LRFS **(C)**, and DMFS **(D)** between TPF and TP groups in stage IVA patients with high SII (SII≥432.48). TPF, docetaxel, cisplatin, and 5-fluorouracil; TP, docetaxel and cisplatin; OS, overall survival; PFS, progression-free survival; LRFS, locoregional relapse-free survival; DMFS, distant metastasis-free survival.

Moreover, SII is a promising factor in predicting prognosis of NPC patients. Therefore, based on the different pretreatment SII levels, we separated patients at stage IVA into low- and high-risk groups. Interestingly, our results revealed that in the high-risk group (SII ≥432.48), TPF showed significantly better OS (*P* = 0.038, **Figure 4A**) and DMFS (*P* = 0.028, **Figure 4D**) than TP, while not applicable for PFS (P = 0.099, **Figure 4B**) and LRFS (P = 0.667, **Figure 4C**). Further analysis was conducted and revealed that no significant survival difference was found in the low-risk group (SII <432.48); however, there were only 16 and 10 cases in TPF and TP groups, respectively, which required larger samples to confirm.

### Univariate and Multivariate Analyses

In our univariate analysis, EBV DNA status, TNM stage, and pretreatment SII were corroborated as potential factors affecting all survival outcomes (**Table 2**). Patients with N0-1 stage were found to have a higher DMFS rate than that of N2-3 (88.3 *vs* 84.6%, *P* = 0.038). And in different IC regimens, the TPF regimen showed greater 5-year DMFS rate (90.2 *vs* 81.3%, *P* = 0.043). Considering the confounding factors, only statistically significant variables in univariate analysis were further researched in multivariate cox regression analysis. As shown in **Table 3**, EBV DNA status and clinical stage were related factors affecting all survival outcomes (*P* < 0.050). Also, pretreatment SII was considered as a related prognostic element for PFS (HR 2.801, 95% CI 1.195–6.565, *P* = 0.018) and DMFS (HR 3.735, 95% CI 1.121–12.441, *P* = 0.032). At the same time, IC regimen (HR 2.182, 95% CI 1.002–4.751, *P* = 0.049) and N stage (HR 4.076, 95% CI 0.962–7.267, *P* = 0.046) can also be used as effective indicators for predicting DMFS in LA-NPC patients.

**Table 2:** Univariate analysis of prognostic factors for LA-NPC patients.

Variables	5-year OS (%)	*P*	5-year PFS (%)	*P*	5-year LRFS (%)	*P*	5-year DMFS (%)	*P*
**Age (years)**		0.494		0.636		0.537		0.298
<45	88.0		72.0		88.7		83.2	
≥45	86.1		74.1		85.1		88.4	
**Sex**		0.800		0.688		0.392		0.624
Female	84.9		70.8		81.0		89.7	
Male	87.6		74.4		88.3		85.4	
**Smoke**		0.533		0.655		0.781		0.500
No	88.4		74.5		85.6		88.0	
Yes	85.0		72.3		87.1		85.1	
**Drink**		0.798		0.599		0.700		0.164
No	86.2		74.7		85.6		89.1	
Yes	87.1		71.4		87.2		83.0	
**EBV DNA status**		0.004		0.000		0.001		0.000
Negative	96.0		93.3		95.5		97.8	
Positive	80.8		59.0		79.4		78.5	
**Tumor classification**		0.393		0.259		0.333		0.535
T1-2	90.3		81.0		90.5		90.5	
T3-4	85.7		71.2		85.1		85.4	
**Nodal classification**		0.289		0.479		0.307		0.038
N0-1	91.6		68.1		78.8		88.3	
N2-3	85.7		73.3		88.0		84.6	
**Clinical stage**		0.000		0.001		0.001		0.000
III	99.0		99.0		99.0		99.0	
IVA	75.0		50.2		75.1		74.3	
**Pretreatment SII level**		0.002		0.000		0.008		0.021
<432.48	100		91.0		95.5		95.5	
≥432.48	80.9		65.5		82.1		82.5	
**IC regimen**		0.154		0.080		0.924		0.043
TPF	89.0		76.8		85.9		90.2	
TP	82.4		68.4		86.9		81.3	

EBV DNA, Epstein-Barr virus DNA; SII, systemic immune-inflammation index; IC, induction chemotherapy; TPF, docetaxel, cisplatin, and 5-fluorouracil; TP, docetaxel and cisplatin; OS, overall survival; PFS, progression-free survival; LRFS, locoregional relapse-free survival; DMFS, distant metastasis-free survival.

**Table 3:** Multivariate cox regression analysis of prognostic factors for LA-NPC patients.

Variables	HR (95% CI)	*P*
**OS**		
EBV DNA status (positive *vs* negative)	6.456 (1.496–7.871)	0.012
Nodal classification (N2-3 *vs* N0-1)	2.167 (0.500–9.391)	0.301
Clinical stage (IVA *vs* III)	9.355 (2.588–14.731)	0.004
Pretreatment SII level (≥432.48 *vs* <432.48)	3.977 (0.709–7.314)	0.073
IC regimen (TP *vs* TPF)	1.880 (0.778–4.545)	0.161
**PFS**		
EBV DNA status (positive *vs* negative)	5.254 (2.242–12.314)	0.001
Nodal classification (N2-3 *vs* N0-1)	0.887 (0.637–1.236)	0.480
Clinical stage (IVA *vs* III)	4.956 (5.898–12.845)	0.001
Pretreatment SII level (≥432.48 *vs* <432.48)	2.801 (1.195–6.565)	0.018
IC regimen (TP *vs* TPF)	1.604 (0.941–2.736)	0.083
**LRFS**		
EBV DNA status (positive *vs* negative)	3.358 (1.162–9.700)	0.025
Nodal classification (N2-3 *vs* N0-1)	0.665 (0.303–1.463)	0.311
Clinical stage (IVA *vs* III)	1.479 (2.477–7.839)	0.004
Pretreatment SII level (≥432.48 *vs* <432.48)	0.665 (0.303–1.463)	0.086
IC regimen (TP *vs* TPF)	1.036 (0.495–2.172)	0.924
**DMFS**		
EBV DNA status (positive *vs* negative)	9.871 (2.332–4.774)	0.002
Nodal classification (N2-3 *vs* N0-1)	4.076 (0.962–7.267)	0.046
Clinical stage (IVA *vs* III)	5.201 (2.769–5.011)	0.010
Pretreatment SII level (≥432.48 *vs* <432.48)	3.735 (1.121–12.441)	0.032
IC regimen (TP *vs* TPF)	2.182 (1.002–4.751)	0.049

EBV DNA, Epstein-Barr virus DNA; SII, systemic immune-inflammation index; IC, induction chemotherapy; TPF, docetaxel, cisplatin, and 5-fluorouracil; TP, docetaxel and cisplatin; OS, overall survival; PFS, progression-free survival; LRFS, locoregional relapse-free survival; DMFS, distant metastasis-free survival; HR, hazard ratio; CI, confidence interval.

### Toxicities

As shown in **Table 4**, no significant difference was found in grade 1–2 toxicities between the TPF and TP groups (*P* > 0.050). Compared with TP regimen, we found that the rate of grade 3–4 leukopenia (40.62 *vs* 36.47%, *P* = 0.038), neutropenia (27.34 *vs* 14.12%, *P* = 0.021), radiation oral mucositis (28.91 *vs* 14.12%, *P* = 0.048), diarrhea (27.34 *vs* 10.59%, *P* = 0.036), and ear damage (14.06 *vs* 10.59%, *P* = 0.046) was higher in the TPF group. All the patients with toxicities were improved after treatment, and no interruption of treatment occurred.

**Table 4:** Treatment-related toxicities between the TPF and TP groups.

Variables	TPF (n = 128)			TP (n = 85)			*P*	
	Grade 0 (%)	Grade 1-2 (%)	Grade 3-4 (%)	Grade 0 (%)	Grade 1-2 (%)	Grade 3-4 (%)	Grade 1-2	Grade 3-4
Leukopenia	20 (15.63)	56 (43.75)	52 (40.62)	24 (28.24)	30 (35.29)	31 (36.47)	0.380	0.038
Neutropenia	28 (21.88)	65 (50.78)	35 (27.34)	33 (38.82)	40 (47.06)	12 (14.12)	0.465	0.021
Anemia	46 (35.94)	78 (60.94)	4 (3.12)	36 (42.35)	47 (55.29)	2 (2.36)	0.583	0.154
Thrombocytopenia	44 (34.38)	82 (64.06)	2 (1.56)	28 (32.94)	56 (65.88)	1 (1.18)	0.435	0.083
Abnormal liver function	46 (35.94)	81 (63.28)	1 (0.78)	26 (30.59)	58 (68.23)	1 (1.18)	0.363	0.215
Abnormal renal function	48 (37.50)	79 (61.72)	1 (0.78)	32 (37.65)	53 (62.35)	0 (0.00)	0.672	0.276
Vomiting	23 (17.97)	90 (70.31)	15 (11.72)	12 (14.12)	59 (69.41)	14 (16.47)	0.574	0.426
Oral mucositis	26 (20.31)	65 (50.78)	37 (28.91)	23 (27.06)	50 (58.82)	12 (14.12)	0.375	0.048
Diarrhea	18 (14.06)	75 (58.60)	35 (27.34)	17 (20.00)	59 (69.41)	9 (10.59)	0.584	0.036
Osteonecrosis	126 (98.44)	2 (1.56)	0 (0.00)	84 (98.82)	1 (1.18)	0 (0.00)	0.746	0.548
Ear (deafness/otitis)	89 (69.53)	21 (16.41)	18 (14.06)	65 (76.47)	11 (12.94)	9 (10.59)	0.147	0.046
Radiation-induced malignancy	0 (0.00)	0 (0.00)	0 (0.00)	0 (0.00)	0 (0.00)	0 (0.00)	–	–

TPF, docetaxel, cisplatin, and 5-fluorouracil; TP, docetaxel and cisplatin.

## Discussion

Due to the special anatomical structure and its sensitivity to radiation, radiotherapy is the main treatment for NPC. And as the IMRT advanced, the local control rate has been improved, while local recurrence and distant metastasis are still the main failure (13). Increasing evidences suggested that IC can promote the eradication of micrometastasis, alleviate clinical symptoms caused in short term, and improve radiosensitivity (14). Furthermore, IC has been confirmed to be effective with LA-NPC in several phase III trials (15) and is widely applied. Hence, IC followed by CCRT is suggested to improve survival benefit in LA-NPC. However, it is quite important to find effective IC regimens with fewer side effects. Currently, studies on IC regimens commonly used in LA-NPC include TPF, TP, PF, and GP (16). Zhao et al. (17) found that compared with PF regimen, both GP and TP regimens could significantly improve DFS and OS, and no severe toxicities occurred. And Peng et al. (18) revealed that for NPC patients receiving a cumulative cisplatin dose (CCD) <200 mg/m^2^, TPF showed better survival than TP and PF, while no significant difference was found in patients receiving a CCD ≥200 mg/m^2^.

At present, TPF is the main regimen for LA-NPC, but accompanied by its long treatment time and adverse toxicities caused by 5-FU, such as myelosuppression and diarrhea. In a previous study on locally advanced head and neck squamous cell carcinoma (19), it was found that the total effective rate of TP regimen was 65.4%. The 3-year PFS rate and OS rate were similar as TPF. What is known to us all, different tumors of the head and neck were included in that study, and the response rate of TP regimen was taken as the main endpoint. Wang et al. (8) further found that TPF (docetaxel 60 mg/m2, cisplatin 25 mg/m2, days 1–3, 5-FU 500 mg/m2, days 1–3) showed similar efficacy compared to TP. No significant difference in 3-year survival outcomes was found (*P* > 0.050) between the two IC regimens. And multivariate analysis in this study also reached the same conclusion; however, the grade 3–4 toxicity in TP group is lower and tolerable. On accounting of the toxicities of 5-FU, patients were given lower dosage, so the potential effect of insufficient dose cannot be completely ruled out. At present, there is still no consensus about the efficacy and safety of the two regimens. Therefore, this paper was conducted to compare the efficacy and toxicity of TPF and TP regimen in LA-NPC, in order to explore the feasibility of alternative TP regimen.

Finally, 213 LA-NPC patients were enrolled in our study. It was found that compared to TP, the TPF regimen showed similar short-term efficacy (total effective rate was 79.7 *vs* 78.8%), and no significance in 5-year OS, PFS, and LRFS (*P* > 0.050), which were consistent with Peng (18) and Wang et al. (8). Variously, in our study, TPF was found to have a higher 5-year DMFS rate (90.2 *vs* 81.3% 750 mg/m^2^, *P* = 0.043), which may be due to the therapeutic benefits of 5-FU. Compared with the study of Wang et al. (8) (5-FU 500 mg/m^2^, days 1–3), the dose in our hospital reached 750 mg/m^2^ (days 1–5) in TPF regimen. Similarly, in the NPC-9901 and NPC-9902 study (20), the dose of 5-FU during CCRT was confirmed to improve DFFS, with an explanation that 5-FU could reduce the risk of disease, and this may also be applicable to the IC phase. Nowadays, the TNM staging system is still considered as a critical factor related to prognosis, and we further analyzed survival differences between patients in stage III and IVA, respectively. Interestingly, the same results were found in stage IVA patients. In previous studies, patients with advanced N category (N2–3) were more prone to distant metastasis (21); in our N category subgroups, fortunately, we observed that the TPF group had a trend in higher 5-year DMFS (*P* = 0.057), which was not applicable in N0-1. One possible statement is that TPF can reduce distant metastases from patients with high metastatic burdens (N2–3). Similarly, Guo et al. (22) found that N3 is an independent prognostic factor for LA-NPC, with poorer survival. These findings are similar to the results of our study, that is, compared with TP regimen, TPF regimen can show better survival in LA-NPC, especially in N2-3 patients. For N0-1 patients, the choice of TP regimen with fewer treatment-related toxicities may be enough.

In recent years, more and more evidences supported systemic inflammation contributed to the biological behavior of tumor cells, such as growth, infiltration, and metastasis (23). SII is associated with poor prognosis of NPC as a new biomarker (10), which is defined as an integration of peripheral platelet, neutrophil, and lymphocyte count. It is a comprehensive and objective tool that integrates three indicators together, and it is simpler and cheaper. Oei et al. (24) revealed that pretreatment SII level was an effective predictor for OS, PFS, and DMFS (*P* < 0.05). In our study, it was also confirmed that pretreatment SII was a significant prognostic factor of PFS (HR 2.801, *P* = 0.018) and DMFS (HR 3.735, *P* = 0.032), which was similar as the previous results. Nevertheless, the optimal threshold of SII level before treatment is not consistent in various studies, which may be related to the baseline characteristics in enrolled patients and reference standards for different instruments, and further prospective research to determine the appropriate cutoff value will be more accurate.

The mechanism of SII affecting prognosis may be related to its components. In inflammatory cells, neutrophils are a part of the tumor microenvironment and are closely related to cancer progression, which can promote the development and metastasis of cancer cells by secreting inflammatory mediators, like TNF and IL-6 (25). Similarly, for lymphocytes, tumor growth can be regulated by secreting cytokines, like IFN-γ and TNF-α. And then, platelets are able to increase the number of tumor cells in circulation and further induce epithelial mesenchymal transformation, thus promoting the extravasation of tumor cells to the metastatic site (26). In addition, some evidence suggests that both neutrophils and platelets can further enhance tumor angiogenesis by secreting vascular endothelial cell factors, like fibroblast growth factor and angiopoietin. Hence, a higher SII, defined as a combination of high neutrophil count, high platelet count, and low lymphocyte count, can promote unlimited proliferation and distant metastasis of tumor cells and contribute to a poor prognosis. As far as we know, the prognostic value of IC regimens based on pretreatment SII and TNM stage in LA-NPC was not reported before. According to ROC curve, the patients in stage IVA with SII≥432.48 was defined high-risk group. Interestingly, our results revealed that in the high-risk group, compared with TP, the TPF regimen showed a superior OS (*P* = 0.038) and DMFS (*P* = 0.028); unfortunately, due to a limited sample size in the low SII group, a consistent conclusion has not been reached. Hence, TPF could be considered as the more effective regimen, particularly in high-risk (IVA combined with SII≥432.48) patients. Furthermore, multivariate analysis showed that IC regimen (HR 2.182, *P* = 0.049) and N stage (HR 4.076, *P* = 0.046) could also be used as effective indicators for predicting DMFS in LA-NPC patients.

About the treatment-related side effects, obviously, combinations of three drugs produce more grade 3–4 toxicities. In our study, we found that compared with TP, the rate of grade 3–4 leukopenia (*P* = 0.038), neutropenia (*P* = 0.021), radiation oral mucositis (*P* = 0.048), diarrhea (*P* = 0.036), and ear damage (*P* = 0.046) were more common in the TPF group, which was consistent as previously reported (8, 27). This difference could be attributed to the anti-tumor therapy of 5-FU, since myelosuppression and diarrhea are the common toxicities.

Whereas, there are also some limitations in this study. First of all, this study is a retrospective analysis in a single center and with a small sample size, which is inevitably accompanied by the deviation of data selection. Second, we only studied SII levels before treatment; a dynamic analysis would be more meaningful. Therefore, further multicenter, large-sample, prospective randomized controlled trials are needed to comprehensively compare the effects of different IC regimens on the efficacy and prognosis in LA-NPC patients.

## Conclusion

In summary, our study revealed that TPF regimen showed a higher 5-year DMFS for LA-NPC patients with stage IVA and N2-3, while TP may be enough for stage III and N0-1. In stage IVA combined with pretreatment SII ≥ 432.48 patients, TPF had higher 5-year OS and DMFS, although grade 3–4 toxicities were more common, but most of them can be tolerable.

## Data Availability Statement

The raw data supporting the conclusions of this article will be made available by the authors, without undue reservation. Requests to access these datasets should be directed to xiongying0604@163.com.

## Ethics Statement

The studies involving human participants were reviewed and approved by Union Hospital of Tongji Medical College of Huazhong University of Science and Technology. The patients/participants provided their written informed consent to participate in this study.

## Author Contributions

XY: topic selection, data collection, and article writing. LZ: image editing. LS: suggestions and amendments to experimental methods and data. GP: article guidance and revision. All authors contributed to the article and approved the submitted version.

## Funding

This work was supported by National Natural Science Foundation of China (Grant No. 82071067).

## Conflict of Interest

The authors declare that the research was conducted in the absence of any commercial or financial relationships that could be construed as a potential conflict of interest.

## Publisher’s Note

All claims expressed in this article are solely those of the authors and do not necessarily represent those of their affiliated organizations, or those of the publisher, the editors and the reviewers. Any product that may be evaluated in this article, or claim that may be made by its manufacturer, is not guaranteed or endorsed by the publisher.
