# Supplementary figures and images for: Comparison of TPF and TP Induction Chemotherapy for Locally Advanced Nasopharyngeal Carcinoma Based on TNM Stage and Pretreatment Systemic Immune-Inflammation Index
Source: Front Oncol. 2021 Sep 20;11:731543. doi: 10.3389/fonc.2021.731543 (PMC8488348; doi:10.3389/fonc.2021.731543)

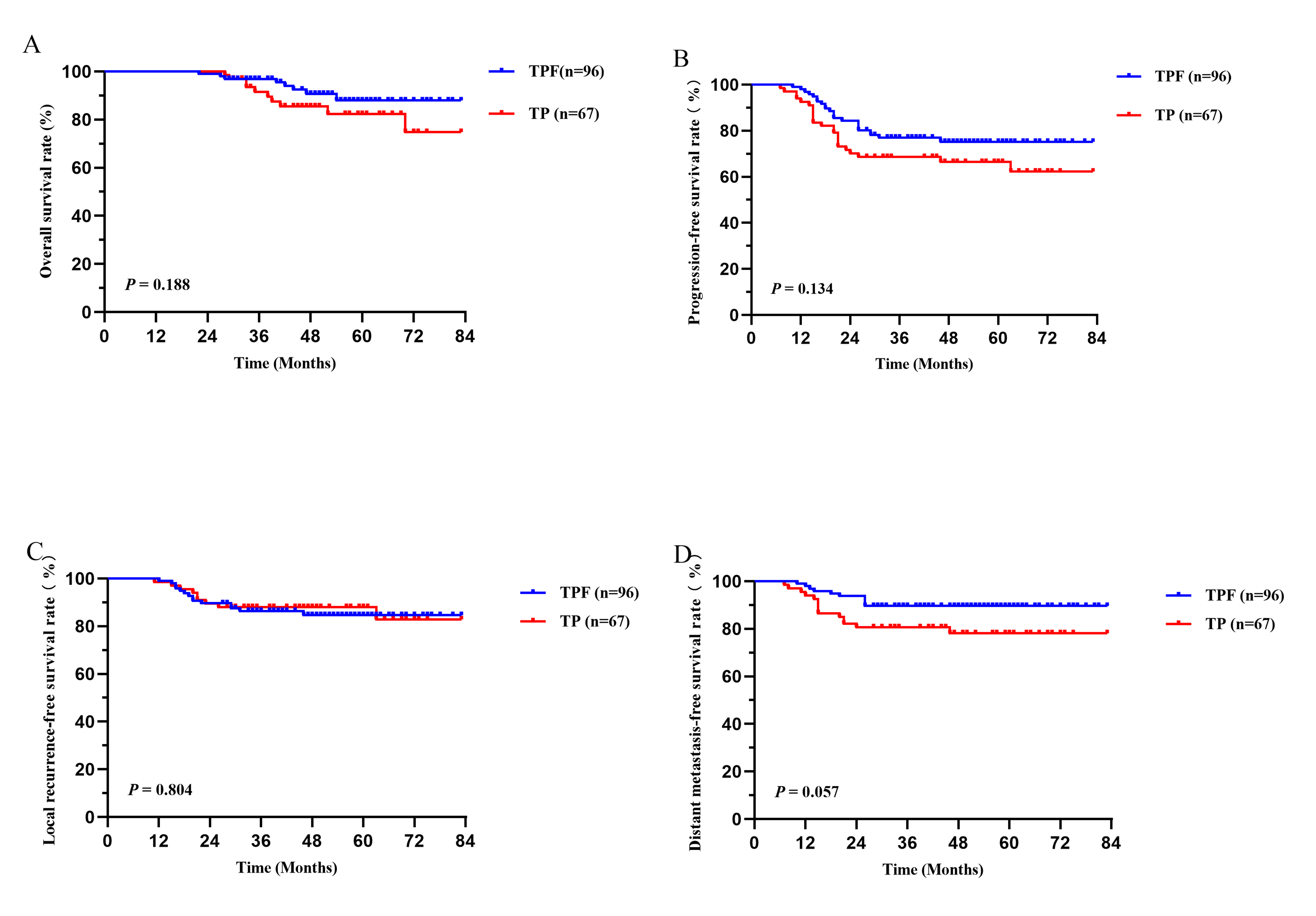

Supplement: Supplementary file 1 [file Image_1.tif]

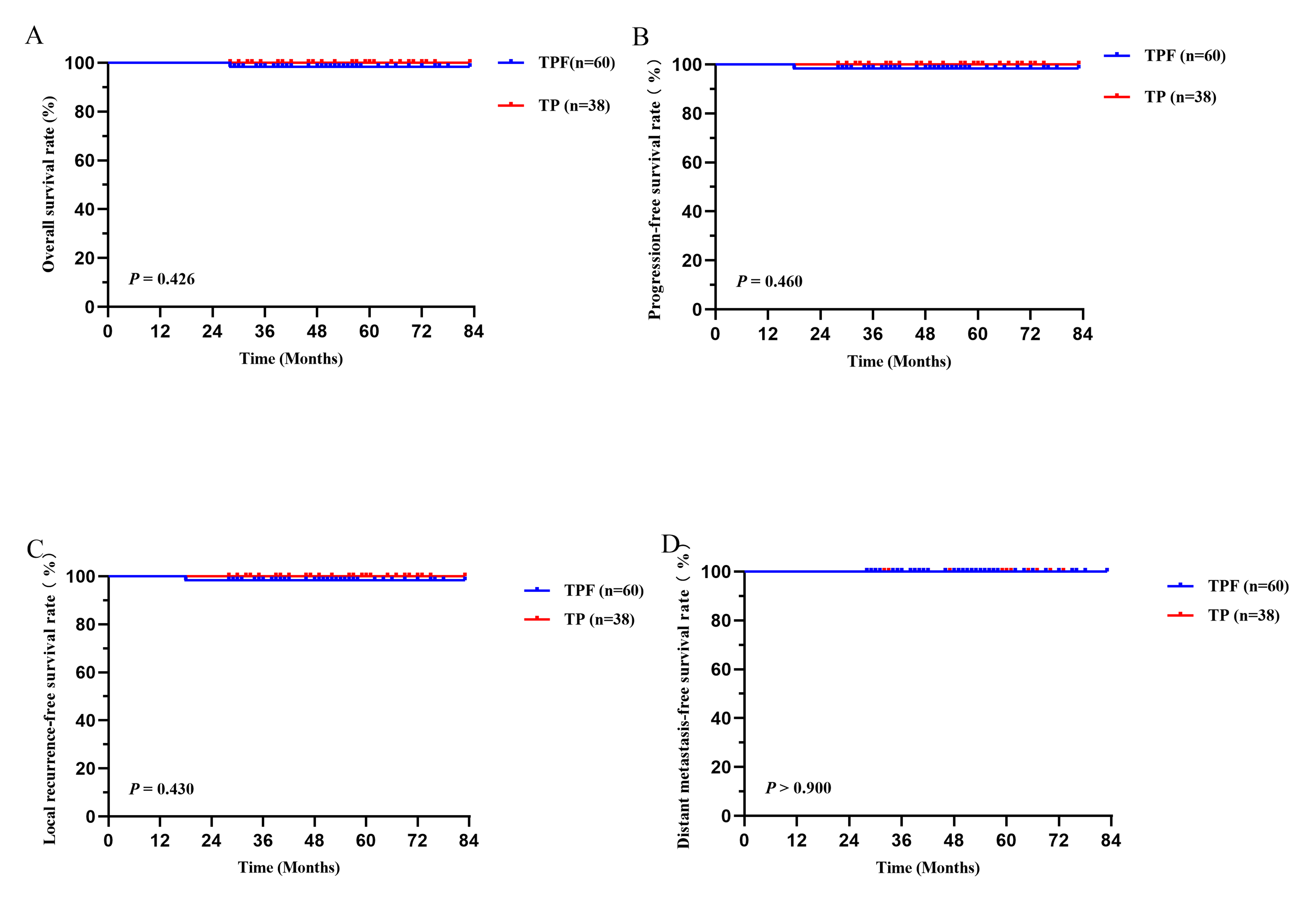

Supplement: Supplementary file 2 [file Image_2.tif]

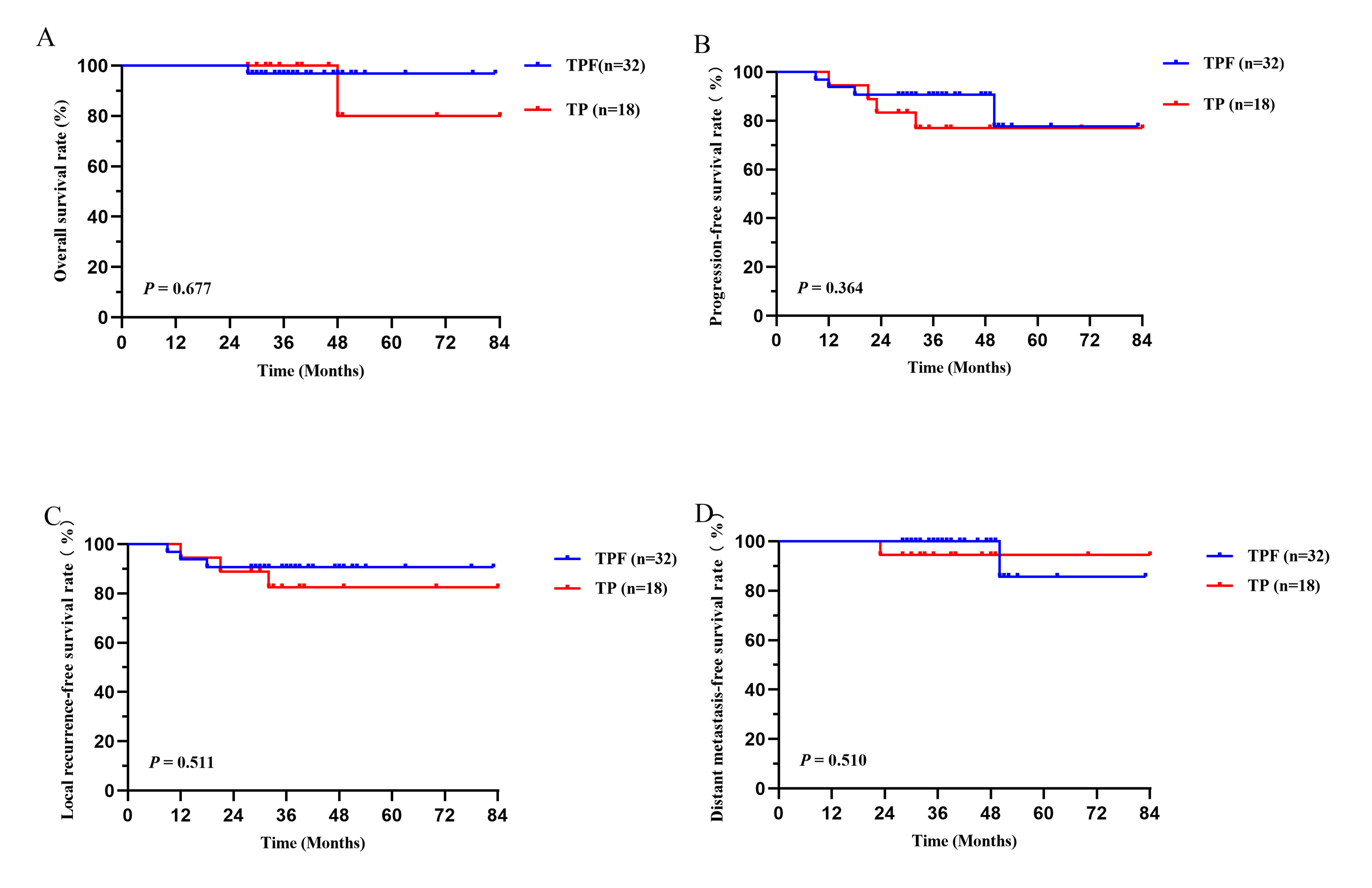

Supplement: Supplementary file 3 [file Image_3.tif]
